# St. John's Wort usage in treating of perinatal depression

**DOI:** 10.3389/fnbeh.2022.1066459

**Published:** 2023-01-05

**Authors:** Rossana C. Zepeda, Claudia Juárez-Portilla, Tania Molina-Jiménez

**Affiliations:** ^1^Laboratorio de Biomedicina Integral y Salud, Centro de Investigaciones Biomédicas, Universidad Veracruzana, Xalapa, Veracruz, Mexico; ^2^Facultad de Química Farmacéutica Biológica, Universidad Veracruzana, Xalapa, Veracruz, Mexico

**Keywords:** *Hypericum perforatum*, pregnancy, postpartum, depression, alternative medicine

## Introduction

Depression is a mental illness characterized by anhedonia, depressed mood, and neurovegetative and neurocognitive symptoms (Malhi and Mann, 2018), causing high disability and affecting the quality of life. Worldwide, about 280 million people suffer from depressive disorders, including major depressive disorder and dysthymia; this number has increased by over 28% with the pandemic (World Health Organization, 2022). Moreover, women are more susceptible to suffering from depression than men. In this regard, the gender gap might be explained by the influence of several factors such as biological (gen-environment interactions, hormonal oscillations, and stress response), psychological (personality traits, interpersonal orientation, and history of disorders), and environmental factors, which involved adversities during early life and sociocultural aspects (Kuehner, 2017). Pregnancy, postpartum, perimenopause, and menstrual cycle are some of the stages of a woman's life associated with vulnerability to developing depression (Cohen, 2003; Kessler, 2003; Soares and Zitek, 2008; Seedat et al., 2009; Schiller et al., 2015; Johann and Ehlert, 2022). For instance, pregnancy is a period in which women undergo several changes regulated by the ovarian hormones, which impact the fetus's development and shape the mother's brain to cope with this new situation (Frokjaer et al., 2015). These physiological changes that occur during pregnancy could also cause biological susceptibility in the mother to developing mental health disorders like depression, i.e., one in seven women who give birth develops postnatal depression (ACOG Committee, 2018). Considering that most of the global population uses herbal medicine because it is their primary source of health care or due to cultural influences (World Health Organization, 2013), it is not surprising that pregnant women prefer to consume natural products without medical supervision because they are perceived as safer for the fetus than allopathic medicine (Wake and Fitie, 2022). Yet, there is not enough evidence for their safety during pregnancy. An example in kind is one of the herbal remedies commonly used for depression: St. John's Wort.

## Perinatal depression

Perinatal depression is defined as an episode of major depressive disorder during pregnancy or after childbirth [American Psychiatric Association (ed.), 2014]. It is estimated that around 10% of pregnant women and women who recently gave birth experience an episode of depression (World Health Organization, 2022). This mental illness affects the mother and the child's emotional and behavioral development, mainly when maternal depressive symptoms persist (Kingston et al., 2018). For example, maternal depression is associated with poor self-care, malnutrition, maladaptive behavior, and pregnancy complications such as a high risk of preterm birth and low birth weight (Jarde et al., 2016). Also, newborns show poor neurobehavioral organization, less responsivity to caregivers and physiological changes, lower activity, cognitive and emotional alterations, and other symptoms related to the lack of mother-infant interaction (Emory and Dieter, 2006; Lefkovics et al., 2014). Thus, early mental health care interventions in obstetrics for pregnant and postpartum women are essential to prevent mother-infant adverse outcomes (Simas et al., 2018).

To this end, a variety of therapies for depression have already been used (Cuijpers and Karyotaki, 2021). For instance, selective serotonin reuptake inhibitors (SSRIs) constitute the first line of pharmacological therapies. SSRIs have shown improvement and remission in women suffering from moderate and severe postnatal depression (Molyneaux et al., 2018; Brown et al., 2021). Because of the risks reported during the development period, it is crucial to consider that this kind of treatment requires personalized care and monitoring to assure its effectiveness and most importantly, the safety and security of the fetus and the newborn (Bałkowiec-Iskra et al., 2017; Mesches et al., 2020).

Nevertheless, pregnancy and breastfeeding are the main reasons why women avoid or discontinue antidepressants, leading to the risk of relapse (Petersen et al., 2011; Payne, 2021). This has led to a search for alternatives, such as traditional medicine, including natural products, and the one that has been among; the most used for treating depression is the St. John's Wort (*Hypericum perforatum* L., Family Hypericaceae).

## Effects of St. John's Wort during pregnancy and postnatal period

*Hypericum perforatum* (HP), known as St. John's Wort, is a plant commonly found in Europe and Asia. It is traditionally used as an alternative therapy to manage depression (Sarris, 2018) and contains many biological compounds, such as flavonoids, hyperforin, and hypericin. These bioactive compounds inhibit the synaptosomal uptake of serotonin, dopamine, and noradrenaline and have affinity for GABAergic and glutamatergic system receptors (Butterweck, 2003). Although HP has shown clinical effectiveness and safety for mild or moderate depression, similar to SSRIs (Apaydin et al., 2016; Ng et al., 2017), some clinical and preclinical studies have reported side-effects and pharmacological interactions associated with HP that might compromise health (Rodríguez-Landa and Contreras, 2003). In this regard, little is known about its effectiveness and safety for treating perinatal depression, which presents a significant health risk since pregnant and breastfeeding women prefer natural products because of their “innocuousness” compared to conventional medicine (Frawley et al., 2015). Thus, it is critical to further study these aspects of HP.

Clinical evidence has shown that HP usage during the first trimester of pregnancy is not associated with malformation, prematurity, or increased rates of stillbirth (Moretti et al., 2009). Nevertheless, some reports indicate that low levels of hyperforin were detected in breast-milk samples from mothers taking HP. Even after the breastfeeding period, infants' plasma levels of hyperforin and hypericin were at the lower limit of quantification. Additionally, no side effects were detected, neither in newborns nor mothers (Klier et al., 2002, 2006). Nonetheless, infants breastfed by mothers taking HP for around 2 months showed colic, drowsiness, and lethargy (Lee et al., 2003). On the other hand, pregnant women consuming HP before the 17th gestational week do not show pregnancy outcomes such as shortened gestational age, preterm birth, abnormal circumference head, low weight, and Apgar scores. However, the authors showed higher rates of malformation in the newborns compared to those detected in the general population (Kolding et al., 2015).

In this sense, data obtained through animal models are controversial (Table 1). On the one hand, rodent females subject to 2 weeks of daily human equivalent doses of HP, or even superior, during gestation and/or postnatal period do not display any behavioral, emotional, and physical long-term outcomes in offspring (Rayburn et al., 2000; Cada et al., 2001). On the other hand, male offspring exposed to HP during pregnancy exhibited less weight at birth. Another study showed that daily HP consumption during gestation, or 2 weeks before conception, did not affect the growth and physical development of mouse offspring (Rayburn et al., 2001). Besides, no toxicity signs were detected in offspring when double human equivalent doses of HP were administered during the 9th to 15th day of pregnancy, the period of organogenesis (Borges et al., 2005).

**Table 1:** Effects of the exposure to SJW and/or its bioactive compounds during the prenatal and postnatal periods in the offspring.

	**Method/test**	**Presentation/extract**	**Source**	**Exposure period**	**Treatment**	**Effect of the treatment/exposure**	**References**
**Human**							
Breastfeeding women	Adverse events	Herbal supplement of SJW	Not mentioned	Lactation	Not mentioned	No adverse effects	Lee et al., 2003
						Isolated cases: colic, drowsiness, lethargy	
Breastfeeding women	Mass spectrometry (LC-MS/MS)	Herbal supplement of SJW (300 mg)	Jarsin^®^, Lichtwer Pharma GmbH, Germany	Lactation	300 mg SJW/3 times daily	HP: ↓ levels/breastmilk	Klier et al., 2002
						↓ Levels/infant plasma (limit of quantification)	
Pregnant women	CES-D, PO	Herbal supplement of SJW (tablets, teas, tincture, or granules)	Not mentioned	Gestation	Pregnant women exposed to SJW	**ne**: depression status, malformation rates, birth and prematurity rats	Moretti et al., 2009
					Depressed women with a disease/conventional drugs		
					Healthy women exposed to teratogens		
					Healthy women		
Pregnant women	Pregnancy outcomes	Herbal supplement of SJW	Not mentioned	17 and 32 gestational weeks	Not mentioned	**ne**: gestational age, preterm birth, head circumference, length, birth weight, and Apgar scores	Kolding et al., 2015
						↑ Malformation incidence	
**Animal model**							
CD1 mouse	EPM/EB/FS/H/LA/ MA/NG/RC/SV/SP	Herbal supplement of SJW (0.3% hypericin)	Basic Organics, Inc., Columbus, Ohio, USA	Gestation/2 w before conception	0.75 mg SJW mixed with each gram of feed	↓ Birth weight of male offspring	Rayburn et al., 2000
						↓ Motor coordination (PND3)	
						**ne**: EB, LA, FS, EPM, SP	
CD1 mouse	BM/PMM/RC	Herbal supplement of SJW (0.3% hypericin)	Basic Organics, Inc., Columbus, Ohio, USA	Mating/gestation	0.75 mg SJW mixed with each gram of feed	↓ Birth weight of male offspring (PND1)	Rayburn et al., 2001
						Delay eruption upper incisors (PND11)	
						**ne**: BM, PMM, RC	
Sprague-Dawley rat	PMM, OFT, ASR, CMT, MWM, EPM	Standardized SJW (0.3% hypericin, 300 mg plant/capsule)	NaturPharma in American Fork, UT, USA	Gestation/lactation	0.18, 0.90, 1.80 or 4.50 g of SJW/kg chow.	↓ Birth weight (PND56/78)	Cada et al., 2001
						**ne:** Brain weight	
						**ne:** OFT, ASR, CMT, MWM, EPM	
Wistar rat	Histological analysis (hematoxylin and eosin staining)	Methanol extract (0.3% total hypericin)	Not mentioned	2 weeks before mating/gestation/lactation	SJW 100 or 1000 mg/k/day	*Pups exposed (100 mg/k/day):* Hepatocyte damage (vacuolization)	Garrovo et al., 2004
						*Pups exposed (1000 mg/k/day):* Hepatocyte: hyaline degeneration/disorganization arrays	
						Lobular fibrosis	
						↓ Glomerular size	
						↓ Bowman's space	
						Hyaline tubular degeneration	
Wistar rat	BM, organs weight, RC	Dry extract of Jarsin (0.4% hypericin)	Galena Quimica e Farmacêutica Ltda, Brazil	Gestation (9th−15th day)	18 mg/kg/twice a day	**ne**: Ambulation, piloerection	Borges et al., 2005
						No diarrhea and deaths.	
						**ne**: Maternal body and organs weight	
						**ne**: Implantation and reabsorption rates and # of fetuses/rat	
Wistar rat	Histologic analysis (hematoxylin and eosin staining)	SJW herb extract	Pharmacy store, SOLGAR Istanbul, Turkey	1 weeks before mating/gestation/ lactation	300 mg/kg in water *ad libitum*	Inflammation, focal necrosis (lobes), deteriorating cell layout in pups' liver	Kahyaoglu et al., 2018
						Hydropic and vacuolar degeneration in fetuses	
						↓ Diameter of glomeruli	
						Bowman capsule distance was absent	
						Hydropic and hyaline degeneration in tubules	
***In vitro* model**							
JEG-3 Cell line	Biological assays	SJW extract standardized (0.3% hypericin) purified hypericin	Homeocan^®^, Montreal, QC, Canada	–	SJW (25–250 mg/mL) Hypericin (7.5 ng/mL and 75 ng/mL)	↓ Viability/JEG-3 cells (SJW 150 and 250 mg/ mL)	da Conceição et al., 2010
			Sigma-Aldrich (St. Louis, MO, USA)				
						**ne**: hCG production (SJW 25 mg/mL or hypericin 7.5 and 75 ng/mL)	
						↑ [Ca2þ]i of JEG- 3 cells (SJW and hypericin)	
						↓ TCTP (SJW)	
						↑ CaBP28K, TRPV-6 (hypericin)	
						↓ PMCA1/4 (hypericin)	
GST-pi/ human placenta at term	EA, PCD	Hypericin	Fluka^®^ (purity: *95 %, HPLC), Germany.	–	Hypericin (0.1, 0.2, 0.4, 60 0.6, 0.8, 1.0, 1.2, 1.6, 3.2, 6.4 mM)	70% inhibition of GST-pi (1 mM hypericin)	Dalmizrak et al., 2012
						Total inhibition of GST-pi (8.5 mM hypericin)	
ES cells NIH/3T3 cell line	Biological assays RT- PCR	Hyperforin	Sigma-Aldrich, St. Louis, MO, USA	–	Hyperforin (0.01–10 mM)	↓ Cell viability/ ES cells, NIH/3T3 cells (dose-dependent)	Nakamura et al., 2013
						Inhibition of ES and NIH/3T3 cell proliferation.	
						↑ Expression Oct4 and Sox2	
						↓ Expression GATA6, TTR, BMP4, NPPA	
åBeWo cell line	Biological assays	*H. perforatum* herb (70% EtOH)	Dixa, St. Gallen Switzerland	–	0.03, 0.1, 0.3, 1, 3, 10, 30, and 100 μg/mL	Cytotoxicity (30-100 μg/mL)	Spiess et al., 2021
						↓ Cell viability	
						↑ Apoptosis (100 μg/mL)	

ANF: ASR, acoustic startle response; BM, body measurements; BMP4, bone morphogenetic protein 4; CaBP28K, cytosolic calcium binding protein; CES-D, center for epidemiologic studies depression scale; CMT, complex maze test; ea, enzymatic assay; EPM, elevation plus maze; EB, exploratory behavior; FS, forced swim; GATA6, GATA binding protein 6; GST-pi, glutathione S-transferase-pi; H, homing; HP, hyperforin; LA, locomotor activity; MA, male aggression; MWM, morris water maze; ne, no effect; NG, negative geotaxis; NPPA, natriuretic peptide type A; Oct4, Octamer-binding transcription factor 4; OFT, open field test; PCD, protein concentration determination; PMCA1/4, plasma membrane Ca^2+^-ATPase 1 and 4; PMM, physical maturation milestone; PND, postnatal day; PO, pregnancy outcomes; RC, reproductive capabilities; SJW, St. John's Wort; Sox2, SRY-box containing gene 2; SV, separation vocalization; SP, social play; TCTP, translationally controlled tumor protein; TPRV6, transient receptor potential; TTR, transthyretin.

Moreover, *in vitro* studies have shown that the ethanolic extract of HP does not modify the placental cell viability, differentiation, and metabolic activity, indicating no cytotoxic or genotoxic effects of HP (Spiess et al., 2021). On the contrary, pups from mothers exposed to HP before mating, during the gestation or breastfeeding period, have focal hepatocyte damage such as hepatocyte hyaline degeneration, fibrosis, and cellular disorganization, which increased when higher doses were used (Garrovo et al., 2004). In the same way, Kahyaoglu et al. (2018) reported that exposure to HP before conception decreases the pregnancy rates and the number of fetuses. In addition, HP exposure during gestation caused an increase in gestation duration and low birth weight. In fact, the histological analysis indicated inflammation in the liver and focal necrosis in each lobe, as well as a morphological alteration in the kidney, possibly caused by oxidative stress, since inducible nitric oxide synthase was elevated in a dose-dependent effect. Moreover, da Conceição et al. (2010) found that HP or hypericin, one of the active compounds, modifies calcium transit in trophoblast-derived cells. It seems that HP and hypericin alter the expression of specific transporters. For instance, HP caused a down-regulation of the Translationally Controlled Tumor Protein (TCTP).

In contrast, hypericin caused a down-regulation of the plasma membrane Ca^2+^-ATPase 1 and 4 (PMCA1/4) and an up-regulation of transient receptor potential (TPRV6) and the cytosolic calcium-binding protein (CaBP28K). These data indicate that HP and its biologically active compounds trigger an imbalance in the Ca^2+^ influx in trophoblast differentiation. Moreover, hypericin competitively inhibits GST-pi, an enzyme that functions in the placenta's detoxification. The latter effect indicates that hypericin could be one of the components contributing to the toxic effects reported in the literature (Dalmizrak et al., 2012), although further work is needed.

## Concluding remarks

Perinatal depression is a common complication caused by vulnerability from all the physiological changes occurring during pregnancy up to the postnatal period. Thus, untreated depression is not an option since adverse health outcomes can affect the mother, the fetus, and even the neonate (Dubovicky et al., 2017). Nevertheless, it is expected that women do not want to use antidepressant drugs before getting pregnant or even drop pharmacological treatment during pregnancy due to the relation between the consumption of antidepressants and the long-term effects on the fetus or neonate (Bałkowiec-Iskra et al., 2017; Dubovicky et al., 2017). Thus, to avoid unnecessary risk to the newborn, they resort to the use of complementary alternative medicine, including natural products like medicinal plants. However, natural medication such as HP is not regulated by high-quality standard processes, unlike allopathic drugs.

Consequently, there is no correct indication of drug interactions, doses, or side effects, to name a few. For instance, HP has around ten components, including hypericin and hyperforin, which appear to be the main ones responsible for its antidepressant effect and its putative effectiveness for mild and moderate depression (Ng et al., 2017). Nevertheless, as Linde et al. (2005) indicate, we have to consider recent studies due to the improvement of trial quality, and the selection of the patient regarding diagnosis.

In this regard, the results must be received with caution, as some evidence mentioned above is contradictory. Some reports indicate that using HP for perinatal depression does not represent a risk to the mother or child. The main active compounds of HP were barely detected in breast milk and the child's serum (Klier et al., 2002). Nevertheless, the participants did not consume the same commercial presentation of the product, and even, they only indicated a standardized extract of HP. The key risk factor is the lack of information about the precise drug concentration or its metabolites, as, in contrast, conventional medications are highly standardized. Moreover, some reports indicate that HP has several drug interactions with other drugs, which interact with P450 cytochrome and P-glycoprotein (Borrelli and Izzo, 2009). For that matter, HP could interact with allopathic antidepressants, increasing their effects and causing a serotonergic syndrome (Izzo, 2004), which could affect the mother and the offspring.

In addition, recent evidence from animal models and *in vitro* studies indicate that HP could cause serious toxicologic effects in the fetus and newborns at doses equivalent to those used for humans and that these toxicological outcomes, such as morphological alterations in the liver and kidneys, are more evident at high doses (Kahyaoglu et al., 2018). In this regard, recent systematic analysis assessing the antidepressant effect of HP during pregnancy and lactation, highlights the limitations of preclinical studies; such as the lack of botanical verification and extract characterization, the ambiguous dosage selection, and the low quality of the methodology and design (Avila et al., 2018), to name a few. Perhaps, the deleterious effects of HP were more prominent in studies in which the experimental procedures were controlled; this allowed to explore modifications at the cellular level and in structural tissues, as well as the expression of essential proteins related to the metabolism of the drugs involved. Overall, it is believed that herbal medicine is safer because it is “natural,” but this is not entirely accurate. Therefore, it is essential to consider that herbal medicine's prescription during gestation or lactation could be unsafe without medical supervision (see Dante et al., 2013). Consequently, more evidence to assure the safety of the consumption of HP as an alternative treatment for perinatal depression is needed to better understand all its pros and cons and make better decisions. To this effect, the main recommendation is the development of obstetrical strategies and programs that offer pregnant women care and support by medical professionals. In the same vein, it is medical professionals who should provide reliable information about the use of herbal treatments such as HP and constant monitoring of the mother and the fetus/newborn to guarantee timely assistance. Finally, the development of regulatory programs to assure safety and effectiveness of herbal medicine constitutes a true challenge that must be addressed.

## Author contributions

TM-J conceived and wrote the original draft. CJ-P and RCZ reviewed the manuscript and provided expertise and feedback.

## References

[B1] ACOG Committee (2018). ACOG Committee Opinion No. 757 summary: screening for perinatal depression, Obstet. Gynecol. 132, 1314–1316. 10.1097/AOG.0000000000002928 30629563

[B2] American Psychiatric Association (ed.). (2014). Guía de consulta de los criterios diagnósticos del DSM-5. Arlington, VA: American Psychiatric Publishing. 10.1176/appi.books.9780890425657

[B3] Apaydin E. A. Maher A. R. Shanman R. Booth M. S. Miles J. N. Sorbero M. E.. (2016). A systematic review of St. John's wort for major depressive disorder. Syst. Rev. 5, 148. 10.1186/s13643-016-0325-2 27589952 PMC5010734

[B4] Avila C. Whitten D. Evans S. (2018). The safety of St John's wort (*H. perforatum) in pregnancy and lactation: a systematic review of rodent studies*. Phytother. Res. PTR 32, 1488–1500. 10.1002/ptr.6099 29708295

[B5] Bałkowiec-Iskra E. Mirowska-Guzel D. M. Wielgoś M. (2017). Effect of antidepressants use in pregnancy on foetus development and adverse effects in newborns. Ginekol. Polska 88, 36–42. 10.5603/GP.a2017.0007 28157249

[B6] Borges L. V. Carmo J. C. D. Peters V. M. Las Casas L. Guerra M. D. O. (2005). Evaluation of *H. perforatum toxicity when administered to pregnant rats*. Rev. Assoc. Medica Brasil. 51, 206–208. 10.1590/S0104-42302005000400016 16127580

[B7] Borrelli F. Izzo A. A. (2009). Herb-drug interactions with St John's wort (*H. perforatum): an update on clinical observations*. AAPS J. 11, 710–727. 10.1208/s12248-009-9146-8 19859815 PMC2782080

[B8] Brown J. V. E. Wilson C. A. Ayre K. Robertson L. South E. Molyneaux E.. (2021). Antidepressant treatment for postnatal depression. Cochrane Database Syst. Rev. 2, CD013560. 10.1002/14651858.CD013560.pub2 33580709 PMC8094614

[B9] Butterweck V. (2003). Mechanism of action of St John's wort in depression: what is known?. CNS Drugs 17, 539–562. 10.2165/00023210-200317080-00001 12775192

[B10] Cada A. M. Hansen D. K. LaBorde J. B. Ferguson S. A. (2001). Minimal effects from developmental exposure to St. *John's wort (H. perforatum) in Sprague-Dawley rats*. Nutr. Neurosci. 4, 135–141. 10.1080/1028415X.2001.11747357 11842881

[B11] Cohen L. S. (2003). Gender-specific considerations in the treatment of mood disorders in women across the life cycle. J. Clin. Psychiatry 64(Suppl 15), 18–29. Retrieved from: https://www.psychiatrist.com/read-pdf/7681/14658987

[B12] Cuijpers P. Karyotaki E. (2021). The effects of psychological treatment of perinatal depression: an overview. Arch. Women's Mental Health 24, 801–806. 10.1007/s00737-021-01159-8 34228202 PMC8492555

[B13] da Conceição A. O. Takser L. Lafond J. (2010). Effect of St. John's Wort standardized extract and hypericin on in vitro placental calcium transport. J. Med. Food 13, 934–942. 10.1089/jmf.2009.0161 20553141

[B14] Dalmizrak O. Kulaksiz-Erkmen G. Ozer N. (2012). Evaluation of the *in vitro* inhibitory impact of hypericin on placental glutathione S-transferase pi. Protein J. 31, 544–549. 10.1007/s10930-012-9433-6 22810152

[B15] Dante G. Pedrielli G. Annessi E. Facchinetti F. (2013). Herb remedies during pregnancy: a systematic review of controlled clinical trials. J. Maternal-Fetal Neonatal Med. 26, 306–312. 10.3109/14767058.2012.722732 22928540

[B16] Dubovicky M. Belovicova K. Csatlosova K. Bogi E. (2017). Risks of using SSRI/SNRI antidepressants during pregnancy and lactation. Interdiscipl. Toxicol. 10, 30–34. 10.1515/intox-2017-0004 30123033 PMC6096863

[B17] Emory E. K. Dieter J. N. I. (2006). Maternal depression and psychotropic medication effects on the human fetus. Ann. N. Y. Acad. Sci. 1094, 287–291. 10.1196/annals.1376.036 17347363

[B18] Frawley J. Adams J. Steel A. Broom A. Gallois C. Sibbritt D. (2015). Women's use and self-prescription of herbal medicine during pregnancy: an examination of 1,835 pregnant women. Women's Health Issues Off. Publ. Jacobs Instit. Women's Health 25, 396–402. 10.1016/j.whi.2015.03.001 25935822

[B19] Frokjaer V. G. Pinborg A. Holst K. K. Overgaard A. Henningsson S. Heede M.. (2015). Role of serotonin transporter changes in depressive responses to sex-steroid hormone manipulation: a positron emission tomography study. Biol. Psychiatry 78, 534–543. 10.1016/j.biopsych.2015.04.015 26004162

[B20] Garrovo C. Rosati A. Bartoli F. Decorti G. (2004). Toxicity of *H. perforatum (St. John's wort) administered during pregnancy and lactation in rats*. Toxicol. Appl. Pharmacol. 200, 201–205. 10.1016/j.taap.2004.04.020 15504456

[B21] Izzo A. A. (2004). Drug interactions with St. John's Wort (H. perforatum): a review of the clinical evidence. Int. J. Clin. Pharmacol. Therapeutics 42, 139–148. 10.5414/CPP42139 15049433

[B22] Jarde A. Morais M. Kingston D. Giallo R. MacQueen G. M. Giglia L.. (2016). Neonatal outcomes in women with untreated antenatal depression compared with women without depression: a systematic review and meta-analysis. JAMA Psychiatry 73, 826–837. 10.1001/jamapsychiatry.2016.0934 27276520

[B23] Johann A. Ehlert U. (2022). Similarities and differences between postpartum depression and depression at other stages of female life: a systematic review. J. Psychosom. Obstetr. Gynaecol. 43, 340–348. 10.1080/0167482X.2021.1962276 34468259

[B24] Kahyaoglu F. Gökçimen A. Demirci B. (2018). Investigation of the embryotoxic and teratogenic effect of *H. perforatum in pregnant rats. Turk*. J. Obstetr. Gynecol. 15, 87–90. 10.4274/tjod.84429 29971184 PMC6022421

[B25] Kessler R. C. (2003). Epidemiology of women and depression. J. Affect. Disord. 74, 5–13. 10.1016/S0165-0327(02)00426-3 12646294

[B26] Kingston D. Kehler H. Austin M.-P. Mughal M. K. Wajid A. Vermeyden L.. (2018). Trajectories of maternal depressive symptoms during pregnancy and the first 12 months postpartum and child externalizing and internalizing behavior at three years. PLoS ONE 13, e0195365. 10.1371/journal.pone.0195365 29652937 PMC5898728

[B27] Klier C. M. Schäfer M. R. Schmid-Siegel B. Lenz G. Mannel M. (2002). St. John's wort (H. perforatum): is it safe during breastfeeding?. Pharmacopsychiatry 35, 29–30. 10.1055/s-2002-19832 11819157

[B28] Klier C. M. Schmid-Siegel B. Schafer M. R. Lenz G. Saria A. Lee A.. (2006). St. John's wort (*H. perforatum*) and breastfeeding: plasma and breast milk concentrations of hyperforin for *5 mothers and 2 infants*. J. Clin. Psychiatry 67, 305–309. 10.4088/JCP.v67n0219 16566628

[B29] Kolding L. Pedersen L. H. Henriksen T. B. Olsen J. Grzeskowiak L. E. (2015). *Hypericum perforatum* use during pregnancy and pregnancy outcome. Reprod. Toxicol. 58, 234–237. 10.1016/j.reprotox.2015.10.003 26536653

[B30] Kuehner C. (2017). Why is depression more common among women than among men?. Lancet Psychiatry 4, 146–158. 10.1016/S2215-0366(16)30263-2 27856392

[B31] Lee A. Minhas R. Matsuda N. Lam M. Ito S. (2003). The safety of St. *John's wort (H. perforatum) during breastfeeding*. J. Clin. Psychiatry 64, 966–968. 10.4088/JCP.v64n0818 12927015

[B32] Lefkovics E. Baji I. Rigó J. (2014). Impact of maternal depression on pregnancies and on early attachment. Infant Mental Health J. 35, 354–365. 10.1002/imhj.21450 25798487

[B33] Linde K. Mulrow C. D. Berner M. Egger M. (2005). St John's wort for depression. Cochrane Database Syst. Rev. 2, *CD*000448. 10.1002/14651858.CD000448.pub2 15846605

[B34] Malhi G. S. Mann J. J. (2018). Depression. Lancet 392, 2299–2312. 10.1016/S0140-6736(18)31948-2 30396512

[B35] Mesches G. A. Wisner K. L. Betcher H. K. (2020). A common clinical conundrum: antidepressant treatment of depression in pregnant women. Semin. Perinatol. 44, 151229. 10.1016/j.semperi.2020.151229 32085857 PMC7214132

[B36] Molyneaux E. Telesia L. A. Henshaw C. Boath E. Bradley E. Howard L. M. (2018). Antidepressants for preventing postnatal depression. Cochrane Database Syst. Rev. 4, *CD*004363. 10.1002/14651858.CD004363.pub3 29669175 PMC6494522

[B37] Moretti M. E. Maxson A. Hanna F. Koren G. (2009). Evaluating the safety of St. John's Wort in human pregnancy. Reprod. Toxicol. 28, 96–99. 10.1016/j.reprotox.2009.02.003 19491000

[B38] Nakamura K. Aizawa K. Yamauchi J. Tanoue A. (2013). Hyperforin inhibits cell proliferation and differentiation in mouse embryonic stem cells. Cell Prolifer. 46, 529–537. 10.1111/cpr.12060 24033566 PMC6495937

[B39] Ng Q. X. Venkatanarayanan N. Ho C. Y. X. (2017). Clinical use of *H. perforatum (St John's Wort) in depression: a meta-analysis*. J. Affect. Disord. 210, 211–221. 10.1016/j.jad.2016.12.048 28064110

[B40] Payne J. L. (2021). Psychiatric medication use in pregnancy and breastfeeding. Obstetr. Gynecol. Clin. N. Am. 48, 131–149. 10.1016/j.ogc.2020.11.006 33573783

[B41] Petersen I. Gilbert R. E. Evans S. J. W. Man S.-L. Nazareth I. (2011). Pregnancy as a major determinant for discontinuation of antidepressants: an analysis of data from The Health Improvement Network. J. Clin. Psychiatry 72, 979–985. 10.4088/JCP.10m06090blu 21457681

[B42] Rayburn W. F. Christensen H. D. Gonzalez C. L. (2000). Effect of antenatal exposure to Saint John's wort (Hypericum) on neurobehavior of developing mice. Am. J. Obstetr. Gynecol. 183, 1225–1231. 10.1067/mob.2000.108889 11084570

[B43] Rayburn W. F. Gonzalez C. L. Christensen H. D. Stewart J. D. (2001). Effect of prenatally administered hypericum (St John's wort) on growth and physical maturation of mouse offspring. Am. J. Obstetr. Gynecol. 184, 191–195. 10.1067/mob.2001.108339 11174501

[B44] Rodríguez-Landa J. F. Contreras C. M. (2003). A review of clinical and experimental observations about antidepressant actions and side effects produced by *H. perforatum extracts. Phytomed*. Int. J. Phytotherapy Phytopharmacol. 10, 688–699. 10.1078/0944-7113-00340 14692732

[B45] Sarris J. (2018). Herbal medicines in the treatment of psychiatric disorders: 10-year updated review. Phytotherapy Res. PTR 32, 1147–1162. 10.1002/ptr.6055 29575228

[B46] Schiller C. E. Meltzer-Brody S. Rubinow D. R. (2015). The role of reproductive hormones in postpartum depression. CNS Spect. 20, 48–59. 10.1017/S1092852914000480 25263255 PMC4363269

[B47] Seedat S. Scott K. M. Angermeyer M. C. Berglund P. Bromet E. J. Brugha T. S.. (2009). Cross-national associations between gender and mental disorders in the World Health Organization World Mental Health Surveys. Arch. Gen. Psychiatry 66, 785–795. 10.1001/archgenpsychiatry.2009.36 19581570 PMC2810067

[B48] Simas T. A. M. Flynn M. P. Kroll-Desrosiers A. R. Carvalho S. M. Levin L. L. Biebel K.. (2018). A systematic review of integrated care interventions addressing perinatal depression care in ambulatory obstetric care settings. Clin. Obstetr. Gynecol. 61, 573–590. 10.1097/GRF.0000000000000360 29553986 PMC6059986

[B49] Soares C. N. Zitek B. (2008). Reproductive hormone sensitivity and risk for depression across the female life cycle: a continuum of vulnerability?. J. Psychiatry Neurosci. JPN 33, 331–343. Retrieved from: https://www.jpn.ca/content/33/4/331.long 18592034 PMC2440795

[B50] Spiess D. Winker M. Chauveau A. Abegg V. F. Potterat O. Hamburger M.. (2021). Medicinal plants for the treatment of mental diseases in pregnancy: an *in vitro* safety assessment. Planta Med. 88, 1036–1046. 10.1055/a-1628-8132 34624906 PMC9519192

[B51] Wake G. E. Fitie G. W. (2022). Magnitude and determinant factors of herbal medicine utilization among mothers attending their antenatal care at public health institutions in Debre Berhan Town, Ethiopia. Front. Public Health 10, 883053. 10.3389/fpubh.2022.883053 35570953 PMC9098925

[B52] World Health Organization (2013). WHO Traditional Medicine Strategy: 2014–2023. Geneva: World Health Organization. Available online at: https://www.who.int/publications-detail-redirect/9789241506096 (accessed October 10, 2022).

[B53] World Health Organization (2022). World Mental Health Report: Transforming Mental Health for All. Geneva: World Health Organization. Available online at: https://www.who.int/publications-detail-redirect/9789240049338 (accessed September 21, 2022).

